# CIRSE Standards of Practice for the Provision of Emergency IR Care on a 24/7 Basis

**DOI:** 10.1007/s00270-025-04321-2

**Published:** 2026-01-08

**Authors:** Andreas H. Mahnken, Caroline Clausen, Otto van Delden, Francesca Iacobellis, Anna Maria Ierardi, Anthony G. Ryan, Stavros Spiliopoulos, Thomas Rodt

**Affiliations:** 1 Diagnostic and Interventional Radiology and Nuclear Medicine, Josef-Hospital, Klinikum der Ruhr-Universität Bochum, Bochum, Germany; 2 Department of Radiology, Copenhagen University Hospital Rigshospitalet, Blegdamsvej 9, 2100 Copenhagen, Denmark; 3 Amsterdam University Medical Centre, Amsterdam, The Netherlands; 4 Department of General and Emergency Radiology, A. Cardarelli Hospital, Naples, Italy; 5 Fondazione IRCCS Ca’ Granda Ospedale, University of Milan, Milan, Italy; 6 Division of Interventional Radiology, Department of Radiology, University Hospital Waterford, Ardkeen, Waterford City, Ireland; 7 Royal College of Surgeons in Ireland, Dublin, Ireland; 8 University College Cork, Cork, Ireland; 9 2nd Department of Radiology, School of Medicine, “ATTIKON” University General Hospital, National and Kapodistrian University of Athens, Athens, Greece; 10 Department of Diagnostic and Interventional Radiology, Klinikum Lueneburg, Lueneburg, Germany

**Keywords:** Emergency, Interventional radiology, Infrastructure, 24/7 service

## Abstract

**Background:**

Interventional radiology provides effective and minimally invasive means of treating a broad variety of emergency conditions such as bleeding, ischaemia or sepsis. Availability of 24/7 emergency interventional radiology care is an important prerequisite for optimal patient care. It is often lifesaving with generally low complication rates and excellent outcomes; however, safe and sustainable provision of interventional radiology care on a 24/7 basis requires efficient organisation and adequate infrastructure.

**Purpose:**

This document will define the minimum structural and organisational standards required for the safe and sustainable delivery of emergency interventional radiology care on a 24/7 basis, depending on the spectrum of emergencies intended to be treated. CIRSE Standards of Practice documents are not intended to impose a standard of clinical patient care, but recommend a reasonable approach to, and best practices for, the provision of care.

**Methods:**

The writing group was established by the CIRSE Standards of Practice Committee and consisted of eight clinicians with internationally recognised expertise in emergency interventional radiology. The writing group reviewed the existing literature on emergency interventional radiology, performing a pragmatic evidence search using PubMed to search for publications in English and relating to human subjects preferably published from 2010 to 2025. The final recommendations were formulated through consensus.

**Results:**

Interventional radiology has a well-established role in the successful management of a broad variety of emergency conditions. This Standards of Practice document provides up-to-date recommendations for the safe and sustainable delivery of 24/7 emergency interventional radiology care.

## Introduction

The CIRSE Standards of Practice Committee established a writing group which was tasked with producing up-to-date recommendations for the Provision of Emergency IR Care on a 24/7 Basis. CIRSE Standards of Practice (SOP) documents are neither clinical practice guidelines nor systematic reviews of the literature. CIRSE SOP documents are not intended to impose a standard of clinical patient care but rather recommend a reasonable approach to and best practices for the delivery of care; this document specifically addresses the provision of emergency IR care on a 24/7 basis.

## Methods

The writing group, which was established by the CIRSE Standards of Practice Committee, consisted of eight clinicians with internationally recognised expertise in emergency radiology and extensive experience with interventional radiology (IR) management in the emergency setting. The writing group reviewed existing literature on IR management of emergency treatment with a focus on outcomes and infrastructure, performing a pragmatic evidence search using PubMed to select relevant publications in the English language and involving human subjects, preferably published from 2010 to 2025. Where clear gaps were evident, older literature was searched and select references included. The final recommendations were formulated through consensus.

## Background

IR plays a major role in treating a variety of medical emergencies including haemorrhage, stroke, aortic dissection/rupture, acute limb ischaemia (ALI) and many more. Embolisation in acute haemorrhage and drainage of septic fluid collections including emergency nephrostomy are the most commonly performed and best-established emergency IR procedures [1]; however, IR is constantly expanding its techniques and the aforementioned procedures only represent a snapshot of the current situation. New emergency IR procedures, such as transcatheter treatment of acute pulmonary embolism, are on the rise and have the potential to lead to an exponential increase in the demand for emergency IR and the establishment of new structures such as PE response teams. It has been estimated that up to one-third of patients requiring an IR procedure present as emergency cases [2], making this group of patients highly important, not only by virtue of medical need but also by proportion of the total number of patients treated by IR. While all patients should have access to emergency IR procedures whenever required, there is a well-documented gap between availability and need for emergency IR, particularly during out-of-office hours (OOH), resulting in attendant risks for patients due to the absence of IR services [2].

IR units vary in size, type of service, service hours, etc., depending on their local environment (e.g. type/services of hospital, proximity to other units, size of population served, etc.). The provision of emergency IR depends on the local requirements and the intended level of clinical service. The goal of this SOP document is to support practice-building in emergency IR by defining the minimum standards of practice necessary to establish a safe and robust emergency IR service depending on the spectrum of emergencies intended to be treated. Typical emergencies that benefit from IR treatment are highlighted.

### Organisation and Staffing in Emergency Interventional Radiology

Organisation and staffing are two key components for the provision of an effective, robust, and sustainable 24/7 emergency IR service; however, the recognition of relevance, regulatory frameworks, availability of resources and the actual shape of service vary greatly between countries, healthcare providers (HCP) and IR groups.

**Fig. 1:**
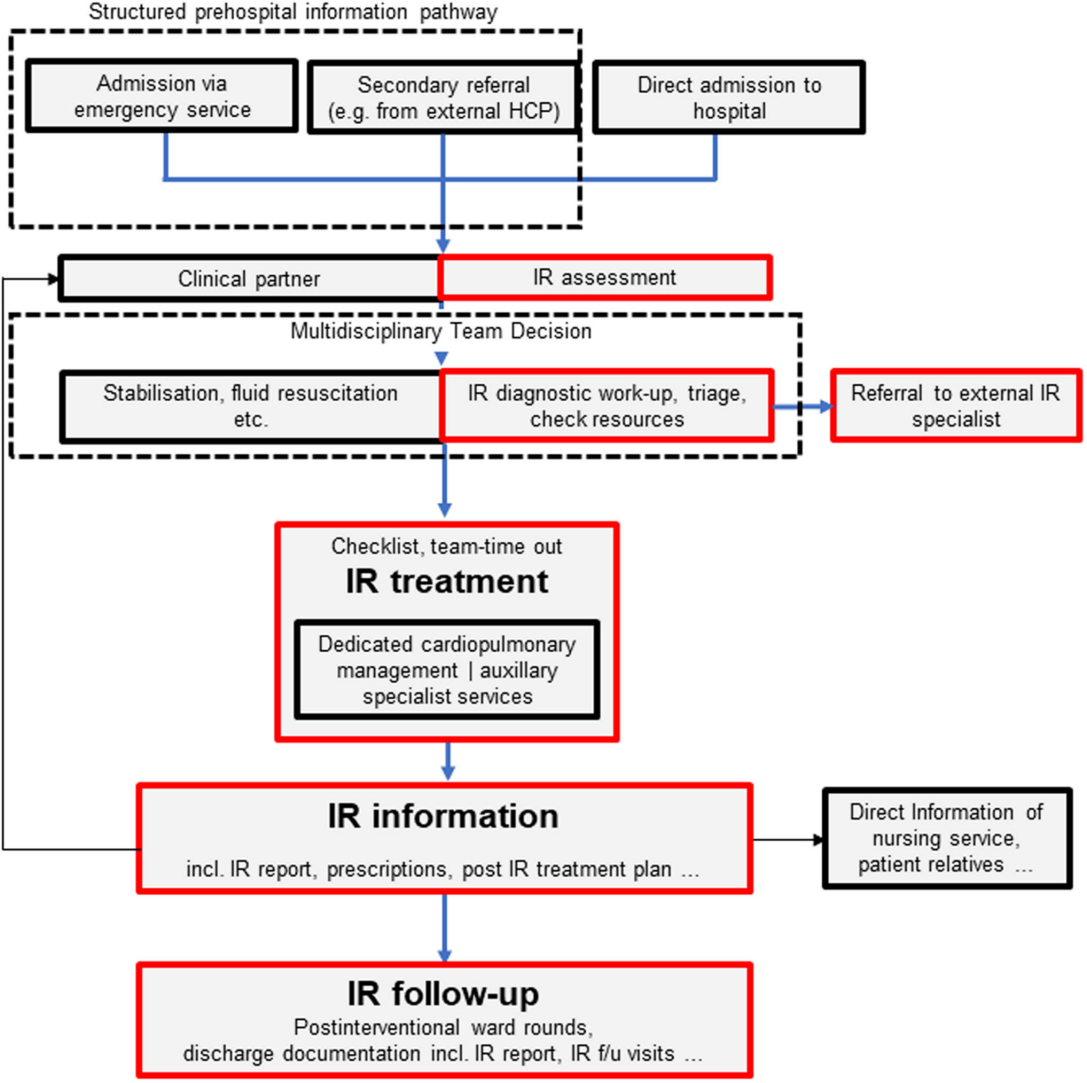
Schematic blueprint of an idealised emergency IR patient pathway. This pathway needs to be adapted for each intended procedure and the individual healthcare providers (HCP) including the relevant local stakeholders

Organisation in this respect implies the strategic planning of different clinical emergency scenarios, taking into consideration the structural circumstances of the respective HCP. This can be achieved by establishing written clinical pathways. Most importantly, IR units should clearly define the emergency procedures they undertake both within working hours and OOH. These pathways should, amongst other things, define the role and responsibility of IR in the clinical workflow in order to ensure IR is notified in a clearly structured way (e.g. by an automated alarm algorithm including all predefined clinical disciplines for a specific scenario) and to predetermine how the ultimate treatment decision is made in cooperation with each medical discipline involved (Fig. 1). A concomitant digital referral pathway and vetting process helps optimise both general and IR workflows in non-life-threatening emergency cases [3]. The development of key performance indicators (KPI) such as CT-to-needle or door-to-treatment-decision is recommended. KPIs may vary between different institutions, but are generally considered essential for quality management and service development.

Institutions should regularly review their internal procedures for development and improvement, taking into account international guidance, local resources and the results of regular internal morbidity and mortality reviews. A summary of key recommendations on the provision of emergency IR care on a 24/7 basis can be found in Table 1 [4, 5].

**Table 1:** Key recommendations for the provision of emergency interventional radiology care on a 24/7 basis

Organisation and Staffing
Define the emergency procedures that are provided both within working hours and OOH
Structured back-up systems such as formal hub-and-spoke arrangements need to be established in case the required service level cannot be provided by the local HCP
Establish written clinical pathways, defining the role and responsibility of IR in the clinical workflow
Establish pathways for 24/7 access to procedure-relevant external imaging studies (e.g. interhospital PACS Systems)
Guidelines, checklists, SOPs, etc., should be adopted and adapted according to the individual HCP’s requirements
For IRs, a minimum 1:6 OOH rota is needed to maintain sustainable services. For populations exceeding 1 million, a 1:8 OOH rota is recommended. Services with 4 or less IRs should cooperate with other IR units
A sufficient number of nurses and/or interventional radiographers needs to be available for an effective and sustainable OOH IR service
The interventional team involved in OOH emergencies should not be involved in diagnostic emergency services at the same time

Technical Equipment
24/7 on-demand access to a CT scanner and uninterrupted access to an IR suite is needed
Secure designated space for essential equipment and supporting specialists such as anaesthesiologists in the IR suite
Advanced cardiac life support equipment must be readily accessible and prepared for use at all times

Patient preparation
Any emergency IR procedures should commence with a multidisciplinary discussion involving all relevant specialties
Adherence to standard operating procedures for planning IR procedures is strongly recommended (e.g. CIRSE IR checklist [4]; CIRSE Standards of Practice on Peri-operative Anticoagulation Management During Interventional Radiology Procedures [5], etc.)
Administer antibiotic prophylaxis before “dirty” non-vascular IR procedures (e.g. nephrostomy, biliary drainage, abscess drainage, cholecystostomy)

*OOH* Out-of-office hours; *HCP* Healthcare providers; *PACS* Picture archiving and communication systems; *SOP* Standards of Practice

The urgency and complexity of emergency IR situations need to be considered in the organisational design of the IR process. Emergencies can be categorised according to clinical triage systems like the ABCDE assessment tool, or the NEWS 2 score [6, 7]. A consistent scoring system should be agreed upon and used for triage and communication when limited resources have to be allocated between different disciplines. The level of complexity is determined by various factors such as altered anatomical conditions, comorbidities, multiple life-threatening conditions, or the need for the simultaneous interdisciplinary treatment of a single condition. Again, mutually agreed internal communication strategies facilitate the management of complex cases.

To ensure the structural groundwork for patient treatment and safety is effective, relevant documents such as guidelines, checklists and SOPs should be adopted and actively adapted according to changing circumstances. The beneficial effects of team training on the provision of emergency IR treatments in terms of process times and staff motivation should be utilised to accompany the more abstract paper-based groundwork [8].

Because of limitations in expertise and infrastructure, procedures may be required that are beyond the scope of the local IR unit. Additionally, the required levels of support from critical care to acute services may not always be available; in these cases, structured back-up systems such as formal hub-and-spoke arrangements with other HCPs must be established.

Numerous back-up systems for stroke, aortic aneurysms or pulmonary embolism have been reported [9, 10]. Typical patterns for back-up arrangements are illustrated below, using the example of stroke treatment:

- drip-and-ship (the patient is brought to a nearby hospital for systemic thrombolysis followed by a transfer to a second hospital for thrombectomy),
- mothership (the patient is brought to a larger, but more distant hospital to receive the entire treatment at the same hospital),
- drip-and-drive/fly (the patient is brought to a nearby hospital for systemic thrombolysis followed by transferal of the interventionalist to the hospital for thrombectomy) [11].

The negative effects of an increased time from presentation to actual treatment due to interhospital transfer are known from the literature [9, 12], and a framework for transferal/repatriation has been described [13]. To further optimise process times for critical emergencies such as stroke and acute aortic syndromes, structured involvement of emergency medical services can facilitate pre-clinical navigation to adequate IR services. For less urgent cases in other diseases, mobile interventional units have been described [14].

Sufficient staffing is another key organisational component for the successful delivery of emergency IR. The existing workforce in both the primary treatment unit and back-up systems has to adequately reflect the workload and distribution of workload over time in a realistic way. It needs to be contracted with the HCP management and documented by obligatory rosters. Minimum staff requirements and the respective level of qualification have been proposed in the literature but may vary depending on individual circumstances [15, 16].

IR is a multidisciplinary specialty involved in direct clinical care. An IR team includes interventional radiologists, radiology nurses, radiographers, and administrative staff, all of whom are needed to provide emergency IR during working hours and OOH. Ideally, the interventional team involved in OOH emergencies should not be involved in providing diagnostic emergency services at the same time. Maintaining appropriate staffing levels and ensuring sufficient rest periods between shifts is essential to maintain sustainable services. It is generally thought that for IRs, a minimum 1:6 OOH on-call rotation (rota) is needed and for populations exceeding 1 million, a 1:8 OOH rota is thought to improve long-term sustainability [2]. Considering the particularly high burnout rate among IRs [17, 18], sustainability is crucial. Consequently, services with 4 or fewer IRs should cooperate with other IR units [15, 19], while teams of six or more IRs are generally capable of delivering a 24/7 service that complies with the European Working Time Directive (EWTD) [2].

Interventional radiographers ensure the production of high-quality images, including advanced imaging techniques such as cone beam computed tomography (CBCT) and fusion imaging while maintaining radiation exposure at the lowest practicable levels [16]. Specialised registered radiology nurses are essential for both patient management and assisting the interventionalist and cannot be substituted by ward nurses with no IR experience. Depending on the complexity of the procedure, at least 1 or 2 nurses and an interventional radiographer are often considered mandatory; however, no valid data exist regarding the number of specialised nurses and interventional radiographers required in emergency IR procedures.

### Technical Equipment Specifications

For 24/7 emergency IR care HCPs need to guarantee unrestricted on-demand access to a CT scanner and uninterrupted access to an IR suite, which should have an area of at least 60m^2^ (74m^2^ for hybrid suites) [20]. Any IR suite requires adequate ventilation air exchange systems, air conditioning and electrical utilities with backup power [15]. For optimal use of the available space, some of the equipment should be ceiling mounted, particularly lighting and some of the radiation-mitigating shielding. This also helps in adjusting the equipment for different procedures. In addition, lighting should be suitable for combined IR and surgical procedures [19].

The core of the IR suite is a C-arm fluoroscopy unit—either ceiling or floor mounted—with a large field-of-view (> 30 cm) detector of adequate resolution capable of advanced imaging, including CBCT and fusion imaging. The use of portable C-arms is discouraged as they are usually unsuitable for handling complex cases. The IR suite should also be equipped with a power injector for contrast administration. Sufficient space should be allocated for both the injector and for warming and preparing the contrast medium. Moreover, the emergency IR suite needs to have direct access to an ultrasound machine including colour duplex ultrasound [21]. Back-up plans are needed in case of technical failure, particularly of the C-arm.

There should be designated space inside the IR suite for storing essential procedural equipment. A minimum of 15 m^2^ for “in-room” supply storage per IR suite is recommended [22]. Sufficient space should be allocated for maintaining an inventory of supplies that may be needed urgently. An additional inventory storage room should be located nearby, e.g. to accommodate additional stock or less commonly used items. Reducing the time spent retrieving supplies leads to shorter procedure times and improved patient safety. It is strongly recommended to establish a structured process to manage the stock of consumables, including storage management, reordering and advancement of the stock to ensure that critical equipment is always available for the emergencies managed by the department.

Advanced cardiac life support equipment must be readily accessible and prepared for use at all times, including monitoring and resuscitation devices [23]. This includes electrocardiogram and blood pressure monitoring equipment, pulse oximeters, an oxygen source along with supplies for its administration, and suction equipment. Wall oxygen and dedicated space for anaesthetic machinery and anaesthesiology staff are essential to deal with more complex emergency IR cases.

A designated space for control room areas and operation of imaging equipment is mandatory. This area should include workstations for image viewing and interpretation, with easy access to picture archiving and communication systems (PACS) and tools to view external CDs [24]. This space may also be used for the storage of radiation safety equipment [25]. The recommended size for this area is at least 36 m^2^.

A scrub area immediately adjacent to the IR suite is needed. Other spaces, e.g. for fluid workflow, patient preparation and recovery around the IR suite are indispensable. For every IR suite, it is recommended to have a minimum of 1.5 beds each for the preparatory and the recovery areas [19]. These areas must be equipped with adequate electrical supply, oxygen, suction and monitoring equipment and should be organised in order to provide privacy to patients [15]. The IR physicians’ room should be in close proximity.

### Patient Preparation

Preprocedural evaluation for emergency IR procedures should always commence with a multidisciplinary discussion involving all relevant specialties during which the treatment goal and plan are clearly defined (Fig. 1). This discussion should include potential contraindications. Although absolute contraindications to emergency IR procedures are rare, especially in life-threatening conditions where alternative treatments are often not feasible, the following aspects need to be discussed: suboptimal interventional treatment option necessitating surgery, unsafe access site and an exsanguinating patient failing to respond to aggressive haemodynamic stabilisation measures. If an IR procedure is indicated, immediate efforts should be made to address relative contraindications, such as severe contrast media allergy or impaired renal function.

Close collaboration with the anaesthesiology team is essential to determine the optimal type of anaesthesia for an IR procedure (general anaesthesia, sedation, or local anaesthesia) and the level of support required based on the patient’s clinical status (e.g. haemodynamic instability necessitating fluid and inotrope therapy, or potential instability during the procedure) [26].

Detailed information about possible treatment options and the specific IR procedure should be provided to the patient, and informed consent should be obtained and appropriately documented. If the patient is unable to provide consent and no family member or legally authorised representative is available, the treating physicians may proceed with the procedure in the patient’s best interests [27].

During pre-procedural work-up, cross-sectional imaging is usually required. Multiphasic CTA is particularly necessary in arterial and variceal bleeding and ischaemic diseases to detect and assess the origin and entity of the active bleeding, or to depict and characterise the vessel obstruction causing ischaemia [28]. Ultrasound may offer valuable additional information in specific clinical scenarios, such as urosepsis, biliary sepsis and ALI. Comparing current imaging with previous studies, if available, is in many cases essential to confirm the urgency and necessity of emergency OOH interventions [26]. Interhospital PACS systems can be invaluable in this regard and are especially recommended for hospitals in a hub-and-spoke arrangement with each other.

It is crucial to confirm the availability of all necessary equipment within the department. Adherence to standard operating procedures, such as the CIRSE IR checklist, facilitates effective pre-procedural planning [4, 29]. As emergency cases usually require procedures of medium or high bleeding or thrombotic risk, standard laboratory work-up is required and should include a complete blood count, a comprehensive metabolic panel (electrolytes, renal and liver function) and a basic coagulation profile (International Normalised Ratio (INR), activated partial thromboplastin time (APTT), platelets and fibrinogen), enabling the prompt correction of abnormal parameters [5]. In cases of suspected acquired coagulation disorders that require specific pharmacotherapy, such as acquired haemophilia A, von Willebrand disease, or Vitamin K deficiency, more advanced testing can be performed [30, 31].

Antibiotic prophylaxis is required before “dirty” non-vascular IR procedures for the prevention of sepsis (e.g. abscess drainage, biliary drainage, cholecystostomy and nephrostomy for pyonephrosis) based on HCP protocols. In general, intravenous antibiotics should be administered within one hour of the procedure. A repeat dose is necessary if two hours have elapsed since the initial dose [32]. Emergency vascular IR procedures (angiography, embolisation, thrombolysis, etc.) can be considered as “clean”, and antibiotic prophylaxis is generally not recommended unless stent-grafts are used or in specific risk patients [32–34].

### Conditions and Treatment

#### Traumatic Arterial Bleeding

Traumatic arterial bleeding amenable to IR treatment is usually the result of blunt trauma and is most often secondary to solid abdominal organ injuries (liver, spleen, kidneys), or pelvic fractures. According to most guidelines, haemodynamically stable or abnormal (but not unstable) patients with abdominal injury can be treated with embolisation. The same holds true for stabilised patients, who may require ongoing anaesthesiology support. In the case of pelvic fracture or if there are no valid surgical options, haemodynamically unstable patients are also amenable to embolisation. It is therefore essential to have appropriate anaesthetic support present in the IR suite to facilitate ongoing resuscitation as required during the procedure.

Almost all patients will be imaged with CT(A) pre-procedurally, although in haemodynamically unstable patients with pelvic fracture, pelvic X-ray and abdominal ultrasound alone may have to suffice [35]. Intraprocedural imaging with DSA showing frank extravasation, false aneurysm, or other indirect signs of vascular injury, such as “cut-off vessels”, is indications for embolisation. Embolisation is most often performed using coils if the patient’s coagulation cascade is intact. Other agents, such as gelfoam or glue, may also be used, particularly in the case of coagulopathy or diffuse bleeding with multiple bleeding foci. Stent-grafts are another effective device to manage arterial bleeding, particularly if there is a major vessel injury. In general, embolisation should be performed as selectively as possible; however, the risks and benefits of the techniques used should be carefully balanced, taking into consideration time-pressure, technical feasibility and complication risk. For splenic injuries in particular, both selective and unselective (proximal) embolisation may be performed safely based on the specific situation. In critical cases Resuscitative Endovascular Balloon Occlusion of the Aorta (REBOA) can be used as a “bridge” to surgery.

The necessity for and timing of follow-up imaging is not standardised; however, it is recommended for non-operatively managed solid organ injuries, and in the presence of clinical signs of rebleeding or other complications requiring additional treatment.

The majority of available data applies to solid organ injuries and pelvic fractures [26, 36].

- Technical & clinical success rates: 73–100%
- Procedure-specific complications: non-target embolisation (ischaemia, necrosis, neurological complications)
- Complication rates: minor: < 10%, major: < 1%

#### Non-Variceal Gastro-Intestinal Bleeding

Non-variceal (or arterial) gastrointestinal (GI) bleeding is usually divided into upper and lower GI bleeding (proximal vs. distal to the ligament of Treitz), with upper GI bleeding accounting for about 85–90% of cases. For upper GI bleeding the first-line treatment is usually endoscopy, unless altered anatomy renders endoscopy impossible or there is bleeding from the pancreas or the bile ducts in which circumstances, embolisation is the first choice. Endoscopy is also often the first-line treatment for lower GI bleeding; however, in haemodynamically unstable patients with lower GI bleeding, embolisation may also be used as first-line treatment [37]. In this setting, CTA is essential to confirm ongoing bleeding and facilitate treatment planning.

Embolisation is indicated when endoscopy fails or bleeding recurs after endoscopy for either upper or lower GI bleeding. Intraprocedural imaging with DSA, ideally with the use of a power injector, showing frank extravasation or false aneurysm are indications for embolisation. In upper GI bleeding, empiric embolisation can sometimes be performed in the absence of angiographic extravasation if the bleeding site is known or strongly suspected from endoscopy or CTA.

Embolisation is usually performed with coils, but other agents such as gelfoam or glue may also be used in special situations, particularly in the presence of coagulopathy. Care should be taken to embolize as selectively as possible to minimise the risk for bowel ischaemia, which is considered higher in the lower GI tract than the upper GI tract. Conversely, the risk for rebleeding is considered higher in the upper GI tract. Because of the rich collateral vascular supply in the upper GI tract, embolisation on both sides of the bleed (“front and back door”) should always be considered. Particular care is required following upper GI surgery as collateral pathways will be disrupted and the risk of ischaemia is higher as a result.

Routine follow-up imaging is not necessary unless there are clinical signs of rebleeding or other complications requiring additional treatment.

There is some variety in the results for upper and lower GI bleeding, e.g. depending on the location of bleeding and the embolics used [38–40].

- Technical success rate: 89–100%
- Clinical success rates: 70–88%; 30-day rebleeding rates: 4.5–33%
- Procedure-specific complications: bowel ischaemia/necrosis (more frequent in lower GI bleeding) and non-target embolisation
- Complication rate: major: 0–9%

#### Variceal Gastro-Intestinal Bleeding

For acute variceal bleeding, transjugular intrahepatic portosystemic shunt (TIPS) creation with or without additional embolisation of varices is indicated [41]. If TIPS is neither possible nor indicated, embolisation of varices alone via a direct percutaneous approach (e.g., transhepatic or transsplenic) can be performed. The most common indications for acute TIPS are variceal bleeding refractory to endoscopic treatment and early and/or recurrent rebleeding after endoscopy. It is important to establish the indication in close cooperation with the hepatologist, taking into account prior history, liver function and the risk for developing hepatic encephalopathy. When performing (acute) TIPS, it is mandatory to have access to deep sedation or general anaesthesia and all methods of resuscitation.

Different techniques for performing TIPS, particularly for guiding the puncture from the hepatic vein into the portal vein, are available. Stenting is performed using dedicated, partly covered TIPS-stents with a controllable diameter. The diameter should be between 7–10 mm based on the portosystemic pressure gradients. In case of ongoing bleeding, concomitant embolisation of varices is usually recommended, which is performed with coils and/or glue/sclerosants. Anticoagulants (heparin) are administrated during the procedure on a case-by-case basis.

There is evidence supporting the use of early TIPS for: oesophageal or gastric variceal bleeding in high-risk patients, persistent oesophageal or gastric variceal bleeding, early oesophageal or gastric variceal re-bleeding and in failed secondary bleeding prophylaxis. Balloon-occluded retrograde transvenous obliteration (BRTO) serves as an alternative to TIPS with comparable efficacy in managing acute bleeding from gastroesophageal varices (Sarin type 2), isolated gastric varices (Sarin type 1), and ectopic varices.

Results vary, e.g. depending on baseline condition, centre experience, etc. [41, 42].

- Technical success rate: shunt dysfunction at 6 and 12 months: 13% and 15–20%
- Clinical success rates: 92–97% freedom from rebleeding; 1-year survival: 78–86%
- Procedure-specific complications: Hepatic encephalopathy, bleeding, sepsis, cholangitis, and pneumothorax
- Complication rates: hepatic encephalopathy 30–50% (pre-TIPS 20%) and other major: 0.6–4.3%

#### Obstetric Emergencies (Post-Partum Haemorrhage)

Post-partum haemorrhage usually results from uterine atony which in most cases can be treated with the aid of uterotonics and intra-uterine balloons; however, this is occasionally insufficient and embolisation is needed. Patients are usually haemodynamically unstable or abnormal and require resuscitation and monitoring with anaesthetic support. Pre-procedural imaging is usually not required and embolisation of both uterine arteries with a resorbable embolic agent (e.g. gelfoam) should be performed regardless of whether contrast extravasation is visible or not. Other types of post-partum haemorrhage, such as bleeding from a caesarean section uterine incision or a significant uterine AV-fistula, may require a different approach with selective embolisation when a focal bleeding point is seen. Embolisation strategies for atypical placentation depend on the type of anomaly. If the patient is adequately stable and emergency MRI is available, a non-contrast MR with a dedicated protocol can be very helpful in selecting the optimal management. If diagnosed antepartum, prophylactic balloon occlusion of the common or internal iliac arteries may be considered. No specific follow-up care or imaging is required after treatment of post-partum haemorrhage.

Outcome varies to some degree, mostly depending on the clinical scenario [43, 44]:

- Technical & clinical success rates: 79–100%; mortality: < 2%
- Procedure-specific complications: non-target embolisation (ischaemia, necrosis, neurological complications, transient ovarian failure).
- Complication rates: minor: 1.5–7%; major: 1.5–18%

#### Acute Limb Ischaemia

The type of intervention performed for ALI in patients with peripheral artery disease (PAD) depends on the type of vascular occlusion (thrombosis vs. embolic event vs. combination) and on the clinical circumstances, including comorbidities and severity of the limb ischaemia. The type of treatment (pharmacologic thrombolysis, mechanic thrombectomy, percutaneous embolectomy or open surgery) should be decided on in close cooperation with vascular surgery. The combination of clinical and imaging findings is essential components when preparing a treatment plan.

Before starting pharmacological thrombolysis, evaluation of all potential contraindications is mandatory. During treatment, a medium- or high-care environment should be available. Appropriate protocols describing thrombolytic doses, intervals between and indications for control angiograms, as well as endpoints and clinical responsibilities need to be in place. Progress should be monitored clinically and angiographically, according to the individual HCP’s standards. For mechanical thrombectomy and percutaneous embolectomy the use of anticoagulants during and after the procedure should also be protocolised and well documented. It is critically important that underlying stenoses and occlusions are immediately treated after thrombolysis or thrombectomy.

Follow-up can be done with cross-sectional imaging or duplex imaging according to local protocol.

Data suggest that intraarterial catheter directed thrombolysis is equivalent to surgery in ALI in terms of thrombotic and embolic events.

- Technical success rate: 80–90%
- Clinical success rates: major amputation free survival 84% after 30 days and 75% after one year [45, 46]; 5-year follow-up without reintervention: 51% [47]; Mortality: 6.7% [48]
- Procedure-specific complications: significant haemorrhage and haemorrhagic stroke
- Complication rates: minor: 0.4–30%; major: 0.4–2.3%

#### Acute Mesenteric Ischaemia (Arterial and Venous)

Arterial and venous mesenteric ischaemia are distinctly different clinical entities requiring different approaches. Thus, a precise diagnosis based on both multiphasic CTA and the clinical severity are crucial for determining the treatment. Mesenteric venous thrombosis accounts for approximately 5% of acute mesenteric ischaemia. Aetiologies of acute arterial mesenteric ischaemia include atherosclerosis, thromboembolic event and least frequently dissection. As the risk for irreversible bowel ischaemia is high, cooperation with surgeons to decide who needs immediate laparoscopy or laparotomy, and who can be treated by percutaneous pharmacological thrombolysis or percutaneous revascularisation is important. In centres where the expertise exists, revascularisation is performed prior to surgery in order to have well demarcated borders of resection. As described above, it is mandatory to have protocols in place describing doses of thrombolytics, frequency of control angiograms, and endpoints when performing pharmacologic thrombolysis for arterial acute mesenteric ischaemia. Percutaneous thrombectomy or embolectomy and stent(-graft) placement can also be performed in acute arterial mesenteric ischaemia and should be accompanied by the appropriate anticoagulant regime, which commonly comprises dual antiplatelet therapy for at least 1 month followed by lifelong antiplatelet monotherapy and anticoagulant therapy for 6 months, or lifelong if required by another condition.

Although the above-mentioned techniques are also used in venous acute mesenteric ischaemia usually via a transhepatic portal approach, there are no universally accepted guidelines defining their role. Anaesthetic support may be required when using a transhepatic approach. Follow-up imaging can be performed with CT and duplex imaging.

Endovascular revascularisation has proven superior to open surgery [49–52].

- Technical success rate: 75–92%
- Clinical success rates: bowel resection: 14–45%; mortality: 15–39%
- Procedure-specific complications: GI bleeding and (potentially fatal) reperfusion syndrome

Mechanical thrombus aspiration and/or catheter directed thrombolysis with or without TIPS is the cornerstones in interventional mesenteric vein revascularisation.

There is substantial variability in results, e.g. depending on thrombus age and volume [53–57].

- Technical success rate: 57.9–100%
- Clinical success rates: 87–100%
- Procedure-specific complications: intraperitoneal haemorrhage from transhepatic or splenic portal access sites.
- Complication rates: minor: 0–36.8%; major: 0–10.5%

#### Ischaemic Stroke

Over the last decade, several hallmark studies have established the role of mechanical recanalisation for the treatment of ischaemic stroke in international guidelines [58, 59]. Mechanical thrombectomy was initially established for treatment of large vessel occlusions in the anterior circulation in the early time window (< 6 h). Subsequently, evidence has been provided for the treatment of patients at a later stage (up to 24 h) and for patients with posterior circulation and large core infarctions [60]. There is a trend towards extending treatment to medium and small vessels or low NIHSS scores, which appears to improve outcomes in more severe strokes with NIHSS scores > 9 [61]. General challenges to be addressed for IR in the treatment of ischaemic stroke are: patient selection, time to reperfusion and the optimal technical strategy.

Preprocedural imaging protocols vary depending on whether the emphasis is on effective use of resources and time (e.g., CCT/CTA only vs. direct to angio/cone beam CT) or on more detailed information regarding the infarct core and penumbra (MRI vs. CT-perfusion). An imaging protocol including CCT/CTA plus CT perfusion is generally accepted to provide the information needed for treatment decision and planning.

General anaesthesia seems to be superior to other anaesthesia techniques [62]. Blood pressure and coagulation management may be needed and are critical when thrombolytic therapy is employed.

With the evolution of stroke thrombectomy, a multitude of interventional techniques have been reported that differ in detail regarding access (femoral vs. radial), device setup (co- or tri-axial; with or without flow arrest), recanalisation techniques (aspiration vs. (stent-) retrieval alone or in combination) and treatment strategies for extra- and intra-cranial atherosclerotic disease and vessel dissection [63]. As there is not a single best approach for the different scenarios, the working group recommends using the various techniques according to the individual interventionalist’s experience. If needed, different techniques such as stent-retrieval and aspiration can be combined in order to achieve the best possible technical success.

Robust evidence supports the use of stent retrievers and/or aspiration techniques in large vessels such as the internal carotid artery, M1 segment, and the basilar artery.

Results vary, e.g. depending on target vessel size, time window, etc. [64, 65]:

- Technical success rate: 58.7–88% (modified treatment in cerebral infarction (mTICI) score)
- Clinical success rates: 32.6–72% (modified Rankin Scale (mRS) score 0–2 at 90 days); Mortality: 9–21%.
- Procedure-specific complications: distal embolisation, embolisation to previously unaffected areas, vessel perforation
- Complication rates: major: 4–6%

Aneurysmal subarachnoid haemorrhage (aSAH) is another condition often amenable to interventional treatment. It clearly fulfils emergency criteria. While ischaemic stroke accounts for 65.3%, and intracerebral haemorrhage for 28.8%, aSAH causes 5.8% of incident strokes. Treatment should be performed in centres with high case volumes [66]. Due to the comparatively low overall incidence of aSAH of 6.1 / 100.000 person-years the subject is not further elaborated in this publication [66, 67].

#### Aortic Dissection, Aortic Aneurysm Rupture and Traumatic Aortic Injury

Ruptured aortic aneurysms require immediate treatment without delay. Currently, endovascular aortic repair (EVAR/TEVAR) is performed whenever feasible. There is a broad variety of different prostheses commercially available, including branched and fenestrated devices for EVAR and TEVAR. Pre-procedural CTA is crucial for treatment planning as anatomic features determine the treatment options. The procedure itself is performed in an OR environment with facilities for anaesthesia, continuous resuscitation and conversion to open surgery. The choice of anaesthesia (local vs. general anaesthesia) and approach (percutaneous vs. cut-down) may vary.

(Thoracic) aortic dissection is initially treated with blood pressure control, pain control and close monitoring. Subsequent TEVAR is performed depending on multiple factors such as aortic dilatation and symptoms.

Traumatic aortic injury—most often a false aneurysm in the distal arch of the thoracic aorta—is treated with TEVAR to prevent delayed rupture. These patients are usually not rendered unstable as a result of the traumatic aortic injury and TEVAR may be placed after other accompanying injuries have been stabilised.

The procedural steps are similar for various indications, and imaging follow-up should be frequent and standardised as there is a high incidence of post-treatment endoleak.

In ruptured ascending thoracic aortic aneurysm, most patients die before or during surgery. In ruptured descending aortic aneurysms, meta-analyses show lower mortality and complication rates for endovascular compared to open repair [68].

Four randomised controlled trials comparing EVAR with open repair in ruptured abdominal aortic aneurysm documented no statistical difference in perioperative, 30-day and 90-day mortality [69–71].

- Technical success rate: 88–99%
- Clinical success rates: Perioperative mortality 17.3–26.4% [72, 73]
- Procedure-specific complications: myocardial infarction, stroke, renal failure, bowel ischaemia, peripheral thromboembolism requiring minor or major amputation, infection, spinal cord ischaemia
- Complication rates: minor: 7–42%; major: 9–29%

#### Septic Abscess Conditions

Sepsis due to intra-abdominal abscess or infected ascites, biloma (see biliary emergencies), thoracic empyema or urinoma (see renal emergencies) requires prompt treatment with antibiotics and source control by percutaneous drainage. Pre-procedural imaging typically with CT allows for diagnosis and treatment planning. Drainage can be performed with CT-guidance, ultrasound guidance or a combination of ultrasound and fluoroscopy guidance depending on anatomic considerations, local expertise and availability. Most procedures can be performed with local anaesthesia alone without the need for anaesthetic support unless patients are unstable and require continuous monitoring. Underlying conditions such as bowel inflammation, perforation, or anastomotic leakage can usually be treated in a second step after sepsis control by percutaneous drainage. As in other drainage procedures, appropriate catheter care should be provided after the procedure including regular flushing with 10–20 ml of sterile 0.9% saline solution at least three times a day. The type and frequency of follow-up imaging depend on catheter output and clinical parameters such as white blood count (WBC), fever and pain. Persistent fever or unchanged WBC after 2 days indicates undrained pus, and a repeat CT scan is warranted.

Percutaneous image-guided drainage of abscesses is a widely used procedure in almost any anatomic location. Sonographic guidance, with or without fluoroscopy, is often first choice; however, for inaccessible areas CT- and less frequently MRI-guidance is used. In general, more easily accessible locations are associated with higher technical success rates, and simpler abscess conditions, e.g. unilocular, are more likely to result in clinically successful outcomes [74–77].

- Technical success rate: 85–100%
- Clinical success rates: 62–100%
- Procedure-specific complications: bleeding, organ injury and pneumothorax
- Complication rates: Minor: 0,9–6,7. Major: 0–1%

#### Biliary Emergencies

Acute cholangitis due to biliary obstruction or stone disease with superinfection constitutes an emergency as it may rapidly progress to sepsis and multiple organ failure. If endoscopic retrograde cholangiopancreaticography (ERCP) is contraindicated or not feasible, percutaneous transhepatic cholangiodrainage (PTCD) should be performed. Thereafter, treatment of the underlying condition can be performed synchronously or using a staged approach. In most situations permanent stents should only be used after treatment of the active infection. When post-operative or traumatic biliary leakage occurs, PTCD does not always have to be performed immediately as adequate drainage of the intraperitoneal biloma with a catheter as close as possible to the site of the leakage may be appropriate. This facilitates delayed PTCD in a more elective setting. In case of biliary leakage, bile ducts are usually not dilated, rendering biliary access significantly more difficult than in cases of obstruction. In acute cholecystitis, percutaneous cholecystostomy is a valid alternative in high-risk patients who are considered poor candidates for surgery. Indications include bridging to surgery as well as definitive therapy [78].

The procedure should be performed with appropriate anaesthetic support. The use of ultrasound as an adjunct to fluoroscopy is helpful. It is recommended that only biliary drainage be performed initially, and to postpone additional procedures such as stone removal to a more elective stage. Antibiotic prophylaxis covering all common upper GI tract pathogens should be administered before the procedure. It is important to provide appropriate catheter care after the procedure and schedule follow-up procedures to treat underlying pathologies such as stones and strictures.

There is some variation in the reported outcomes with slightly lower success rates for non-dilated bile ducts and transplanted livers [79–81]:

- Technical success rate: 78.3–100%
- Clinical success rates: 68.7–100%
- Procedure-specific complications: bleeding, sepsis, bile peritonitis, bile duct stenosis and pneumothorax
- Complication rates: minor: 2–12%; major: 0.8–5%; mortality: 0–36%

#### Renal Emergencies

Pyonephrosis and acute pyelonephritis due to obstruction or renal stone disease constitute emergencies that may rapidly progress to sepsis and multiple organ failure. Therefore, these should be treated promptly with antibiotics and drainage. Based on the clinical situation, anatomy, underlying disease and local expertise, the choice of treatment (percutaneous nephrostomy or double-J insertion) should be decided on in close cooperation with the urologist. Although no strict guidelines exist on the first choice of treatment, nephrostomy has higher technical and clinical success rates than double-J placement. It can be performed under sonographic, fluoroscopic or CT-guidance. In cases of urinary leakage, an appropriate first step can be to adequately drain the urinoma as close as possible to the site of leakage and perform nephrostomy (usually in a non-dilated collecting system) in a more elective setting.

Percutaneous nephrostomy is generally well tolerated under local anaesthesia alone. Sedation is not usually necessary; however, anaesthetic support can be helpful in septic patients. It is important to provide appropriate catheter care after the procedure and schedule follow-up procedures to treat underlying pathologies such as stones and strictures in an elective setting.

Technical and clinical success rates are excellent with an acceptable safety profile [82–84]

- Technical and clinical success rate: 96–99%
- Procedure-specific complications: bleeding, organ injury and pneumothorax
- Complication rates: minor: 10–28%; major: < 6%

## Conclusion

As many as one-third of all IR procedures are for emergencies. Emergency IR covers a wide and heterogeneous range of procedures that are beneficial and often life saving for patients. The absence of a 24/7 availability of emergency IR puts patients at risk. Therefore, a 24/7 emergency IR service should be a priority for all acute HCPs. Organisation of emergency IR infrastructure and processes is most important; this includes defining procedures that are available 24/7, having well-defined interdisciplinary consented pathways with written SOPs, and clear onward referral pathways including hub-and-spoke arrangements if needed. Provision of a 24/7 emergency IR service is part of any HCP’s direct clinical care. It requires a minimum infrastructure in terms of space, equipment and staff, including interventional radiologists, radiographers, nurses and administrative staff. Appropriate staffing is of particular importance and needs to be adequate to maintain a sustainable service.

## Abbreviations

ALI: Acute limb ischaemia
APTT: Activated partial thromboplastin time
aSAH: Aneurysmal subarachnoid haemorrhage
BRTO: Balloon-occluded retrograde transvenous obliteration
CBCT: Cone beam computed tomography
ERCP: Endoscopic retrograde cholangiopancreaticography
EVAR: Endovascular aortic repair
EWTD: European Working Time Directive
GI: Gastrointestinal
HCP: Healthcare providers
INR: International normalised ratio
IR: Interventional radiology
KPI: Key performance indicator
mTICI: Modified treatment in cerebral infarction
OOH: Out-of-office hours
PACS: Picture archiving and communication systems
PAD: Peripheral artery disease
PTCD: Percutaneous transhepatic cholangiodrainage
REBOA: Resuscitative Endovascular Balloon Occlusion of the Aorta
SOP: Standards of Practice
TEVAR: Thoracic endovascular aortic repair
TIPS: Transjugular intrahepatic portosystemic shunt
WBC: White blood count
